# Seasonal Fluctuation of Polycyclic Aromatic Hydrocarbons and Aerosol Genotoxicity in Long-Range Transported Air Mass Observed at the Western End of Japan

**DOI:** 10.3390/ijerph17041210

**Published:** 2020-02-13

**Authors:** Takashi Kubo, Wenzhi Bai, Masaki Nagae, Yuji Takao

**Affiliations:** 1 Faculty of Environmental Science, Nagasaki University, Nagasaki 852-8521, Japan; 2 Graduate School of Fisheries and Environmental Sciences, Nagasaki University, Nagasaki 852-8521, Japan

**Keywords:** polycyclic aromatic hydrocarbons, genotoxicity, transboundary pollution, seasonal fluctuation, pollution sources

## Abstract

In order to clarify the level transboundary air pollution caused by polycyclic aromatic hydrocarbons (PAHs) and genotoxic substances, aerosols were collected from forest and suburban sites in Nagasaki, west Japan, for 6 years. The PAH concentration was measured, and the genotoxicity of the substances were evaluated using the umu test. The results showed no notable trends in the concentration or toxicity of either sites throughout the study period. The suburban and forest sites shared similar seasonal fluctuation patterns and quantitative values, suggesting that the western end of Japan might be affected by long-range transported pollutants, especially in winter. PAH concentration and genotoxicity showed the same seasonal patterns of increased levels in winter and lower levels in summer. This suggests that PAHs and genotoxic substances were correlated and share common sources. Back trajectory and source analyses were conducted using the diagnostic ratios of PAHs. It was predicted that air pollution by PAHs at the forest site arise predominantly as a result of biomass or coal combustion in continental regions, such as northern parts of China and the Korean Peninsula. This is particularly expected in winter. Therefore, genotoxic substances would also be strongly influenced by transboundary pollution from the continental region. In addition, it was estimated that the contribution of transboundary PAH pollution could reach 70% at the suburban site in winter.

## 1. Introduction

Polycyclic aromatic hydrocarbons (PAHs) are typical pollutants that are unintentionally generated, mainly due to incomplete combustion of organic matter. In addition, since they are present in minute amounts in crude oil and petroleum, they are also released into the environment when these substances leak. Many PAHs have been reported as carcinogens and/or genotoxicants. In particular, benzo[a]pyrene (BaP) is a well-known carcinogen. The International Agency for Research on Cancer (IARC) classifies BaP as Group 1 (carcinogenic to humans). Several other PAHs are classified as Group 2A (probably carcinogenic to humans) and 2B (possibly carcinogenic to humans) [1]. Genotoxicity has been quantitatively evaluated for some PAHs. While metabolism reduces the toxicity of many chemicals, it has been reported that PAH genotoxicity tends to be enhanced after reaction with metabolic enzymes [2].

Up until the present, PAHs have been detected in various environmental samples such as river water [3,4,5], sediment [6,7,8], soil [9,10,11], and airborne particles [12,13,14], which indicates the spread of PAHs pollution. Various environmental emission sources have also been investigated, such as waste water [15,16,17], exhaust gas [18,19,20], biomass burning [21,22,23], and petroleum-related products [24,25,26]. Attempts have also been made to estimate emission sources from the measured concentration ratio of PAHs in the environmental samples [27,28,29].

Environmental pollution is not always restricted to the vicinity of the pollutants’ emission sources. It can create challenges thousands of kilometers away, across the border. This so-called transboundary pollution is an old and new environmental problem. The trail smelter case that began in the 1920s, which led to the establishment of international environmental laws, was caused by transboundary air pollution [30]. The philosophy of “states have the responsibility to ensure that activities within their jurisdiction do not cause damage to the environment of other states” has been shared internationally. It has been affirmed repeatedly in international declarations and conventions, such as the Rio Declaration on Environment and Development [31] and the United Nations Framework Convention on Climate Change [32]. River pollution [33,34,35], acid rain [36,37,38], and aerosols [39,40,41] have been related to transboundary pollution. Since the late 1970s, investigations into PAH-related transboundary air pollution have also been reported [42].

PAHs exist as particulates or gaseous molecules in the atmosphere. It has been reported that some PAHs having four benzene rings, and all PAHs having more than four benzene rings exist in the particulate phase [43]. The concentrations of PAHs in the atmosphere have been measured in various regions, including Europe and Asia [44,45,46]; some of these investigations have indicated the probability of transboundary pollution [47,48,49]. Hayakawa et al. not only measured atmospheric PAHs across Japan for a long time [50,51,52], but also took measurements in foreign continents, including Africa [53,54,55]. Although some studies have compared PAHs by seasons [56,57,58], few studies have considered data over several years in combination with toxicity assays.

Genotoxicity is closely related to carcinogenicity. The Ames mutagenicity test is commonly used for evaluating the genotoxicity of environmental samples [59,60,61]. In recent years, however, there has been increased use of the umu test for testing DNA damage, particularly in aquatic environments [62,63,64]. Few studies have used the umu test to investigate the atmosphere, including seasonal changes in atmospheric genotoxicity. There seems to be no longitudinal studies on the use of umu tests in conjunction with PAH measurements. Unlike PAHs, no methods exist for using umu test results to estimate the source of genotoxicity. Therefore, there are few detailed reports on the origin of genotoxic substances in the atmosphere.

As mentioned above, since some PAHs are well-known carcinogens, their environmental behavior needs to be better understood. In addition to this, many things relating to genotoxic substances in the atmosphere remain unexplained, including their sources. If PAH measurements and umu tests are performed on air samples which have been collected at sites with different anthropogenic effects, their characteristics and sources could be estimated from their long-term fluctuation patterns. The objective of this study was to quantify seasonal fluctuation patterns and trends over several years using PAH measurements and umu test assays on airborne particles that were collected in forest and suburban sites. This was in order to gather information on transboundary pollution and its sources.

## 2. Materials and Methods

### 2.1. Sample Collection

**Figure 1:**
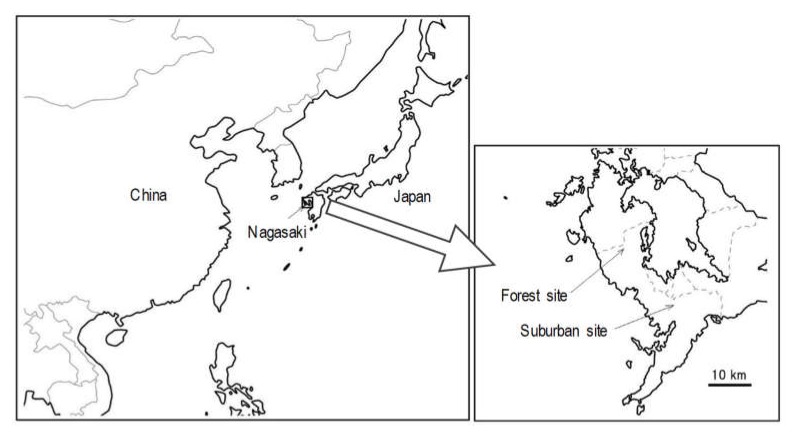
Location of the sampling sites.

The aerosol particles were collected at the forest site and at the suburban site in Nagasaki, Japan, as shown in Figure 1. Nagasaki is located on the western end of Japan. The forest site (32°54′32.4″ N, 129°44′33.5″ E, 533 m above sea level) was in Nagasaki Prefectural Forest Park (18 km northwest from Nagasaki city center), near the top of the Miharashi mountain in Nishisonogi peninsula and covered by trees. The peninsula facing the East China Sea has steep terrain, few flats, and low population density. The site is comparatively isolated from anthropogenic sources of pollution. The suburban site (32°47′07.9″ N, 129°51′52.6″ E, 20 m above sea level) was located at Bunkyo Campus, Nagasaki University. Sampling was conducted on the roof of a four-storied campus building. The campus was surrounded by residential and business areas in Nagasaki city, which has a population of 0.4 million people. The suburban site is 5 km north of the industrial Nagasaki Port area.

Quartz fiber filters (254 mm × 203 mm, QR-100, Advantec, Tokyo, Japan) were preheated at 800 °C for 8 h and weighed after drying in a desiccator for >24 h. The quartz fiber filters were used for collecting aerosols with a high-volume sampler (HV-7000F, Sibata Scientific Technology Ltd., Saitama, Japan). The total suspended aerosol particles were collected, without size selection, by operating the high-volume sampler at a flow rate of 700 L/min. Collection of the aerosol particles were carried out three or four times in each season from 2012 to 2018, in spring (March–May), summer (June–August), fall (September–November), and winter (December–February). The sampling time was 1 week. Rainy days were avoided as much as possible when selecting sampling periods.

### 2.2. PAHs Analysis

Fifteen types of PAHs, including acenaphthylene (Acy), acenaphthene (Ace), fluorene (Flu), phenanthrene (Phe), anthracene (Ant), fluoranthene (Flt), pyrene (Pyr), benzo[a]anthracene (BaA), chrysene (Chr), benzo[b]fluoranthene (BbF), benzo[k]fluoranthene (BkF), benzo[a]pyrene (BaP), dibenz[a,h]anthracene (DahA), indeno [1,2,3-cd]pyrene (IcdP), and benzo[ghi]perylene (BghiP) were selected for quantification in this study with reference to the 16 Priority Pollutant PAHs, as designated by the Environmental Protection Agency (EPA), excluding naphthalene. These standard reagents, apart from Ant, were purchased from AccuStandard, Inc. (New Haven, CT, USA). The Ant was purchased from Nacalai Tesque, Inc. (Kyoto, Japan). Acenaphthene-d_10_, phenanthrene-d_10_, pelyrene-d_12_, and pyrene–d_10_ were used as surrogate compounds for precise measurement. The former three were purchased from Wako Pure Chemical Industries, Ltd. (Osaka, Japan). Pyrene–d_10_ was purchased from Kanto Chemical CO., Inc. (Tokyo, Japan). Silica gel C-200 was purchased from Wako Pure Chemical Industries, Ltd. for cleanup, and the analytical-grade solvents were purchased from Nacalai Tesque, Inc.

Filters were kept in a desiccator for several days, and then the filters were weighed and stored at −20 °C until extraction. The filter was chopped and put in a centrifuge tube, and then 100 ng of each of the four aforementioned surrogates (internal recovery standards) were added to it. Thirty milliliters of acetone was added to this, and ultrasonic extraction was performed for 10 min. After centrifugation, the extract was filtered through a cartridge with a pore size of 0.45 μm. This operation was repeated three times. The filtrate was concentrated to about 1 mL by nitrogen blowing. The solution was fractionated using 5% H_2_O deactivated silica gel column chromatography. PAHs were eluted with 50 mL of an acetone-dichloromethane mixture (3:2, *v*/*v*).

The eluted solution was dehydrated using anhydrous sodium sulfate and concentrated to 1 mL for analysis by nitrogen blowing. PAHs were determined with a 7890A gas chromatograph (Agilent Technologies, Santa Clara, CA, USA) equipped with a 7000A mass spectrometer (Agilent Technologies). One microliter aliquots were injected onto an HP-1MS capillary column (30 m × 0.25-mm i.d., 0.25 μm) with 1.4 mL/min hexane gas. Injector temperature was kept at 300 °C. The column temperature was programmed as follows: 60 °C for 1 min, heat to 300 °C at 10 °C/min, and hold for 5 min. Quantification was conducted in selected ion monitoring (SIM) mode using the molecular weight of each PAH and deuterium-labelled PAH.

### 2.3. Umu Test

After collecting airborne particles, the filter was cut into small pieces, placed in a centrifuge tube and ultrasonically extracted with 30 mL of acetone for 10 min. Extraction, centrifugation, and filtration were repeated, as in the PAH analysis. Then, after the filtrate was almost dried by nitrogen blowing, 500 μL of Dimethyl sulfoxide (DMSO) was added to prepare the umu test solution.

The umu test performed as per the ISO 13829 with slight changes in the overnight culture conditions and the DMSO concentration of the test solution [65,66]. In this umu test using microplates, the test bacteria (*Salmonella typhimurium* TA1535/pSK1002) was brought into contact with a test solution to cause DNA damage. Then, the enzyme induced during the DNA repair processes was reacted with the chromogenic substrate. The intensity of DNA damage was measured from the amount of color development. The bacterial strain was provided by the Environmental Safety Management Laboratory, Faculty of Environment and Information Sciences, Yokohama National University, Japan. The S9 enzyme, obtained from Oriental Yeast Co., Ltd. (Tokyo, Japan), was used to determine genotoxicity in the presence of metabolic activation. The composition of S9 solution was 1.1% to the test solution described in the previous paragraph. The tests without and with the addition of the S9 solution are termed as the “−S9 test” and the “+S9 test”, respectively. Positive control tests were performed each time. As a positive control substance, 4-nitroquinoline-1-oxide (4NQO) was used in the −S9 test, and 2-aminoanthracene (2AA) was used in the +S9 test. The tests were performed using four dose levels in triplicate for each level.

Induction ratio (IR) values were calculated following the ISO 13829. A scatter graph of added amount of air sample (horizontal axis) was plotted against the IR (vertical axis). The slope of the regression line was defined as genotoxicity. To correct for variations in the test sensitivity, genotoxicity was divided by the results of the positive control tests and represented as positive control equivalents, as previously reported [67,68].

### 2.4. Data Analysis

#### 2.4.1. Back Trajectory Analysis

In order to grasp the long-range transport of air masses, back trajectory analysis is performed in many studies [39,40,41]. In this study, we used HYSPLIT Model-Trajectory Frequencies of the National Oceanic and Atmospheric Administration, USA [69,70]. Results from this model indicate the frequency that the trajectory passed over a grid cell. To investigate the typical transport behavior of air mass in each season, the analysis was conducted for 32 days in the middle of each season. This was the maximum number of days that could be set. The main analysis conditions were as follows: forest site source location; 32 days allowed to calculate trajectory frequencies; trajectory frequency grid resolution was set to 2.0 degree; trajectory starting interval was set to 12 h; and total run time was set to 72 h.

#### 2.4.2. PAH Source Identification

Since relationships are thought to exist between the type of source and the PAH composition, the source’s characteristics were estimated from the diagnostic ratio of PAHs. For the estimation, various diagnostic ratios have been proposed [27,28,29]. In this study, Flt/(Flt+Pyr) and IcdP/(IcdP+BghiP) were selected as indicators that use PAHs with a relatively large number of rings and can distinguish three types of sources, namely, petroleum, petroleum combustion, and biomass/coal combustion. For example, when PAH Flt/(Flt+Pyr) levels are <0.4, the PAHs are thought to have petroleum sources; levels of 0.4–0.5 are thought to have come from liquid fossil fuel combustion pollution; and levels >0.5 indicate the grass, wood or coal combustion sources. When PAH IcdP/(IcdP+BghiP)) levels are <0.2, the PAHs are thought to have petroleum sources; levels of 0.2–0.5 are thought to have come from liquid fossil fuel combustion pollution; and levels >0.5 indicate the grass, wood or coal combustion sources [71,72].

## 3. Results and Discussion

### 3.1. Back Trajectory Analysis

**Figure 2:**
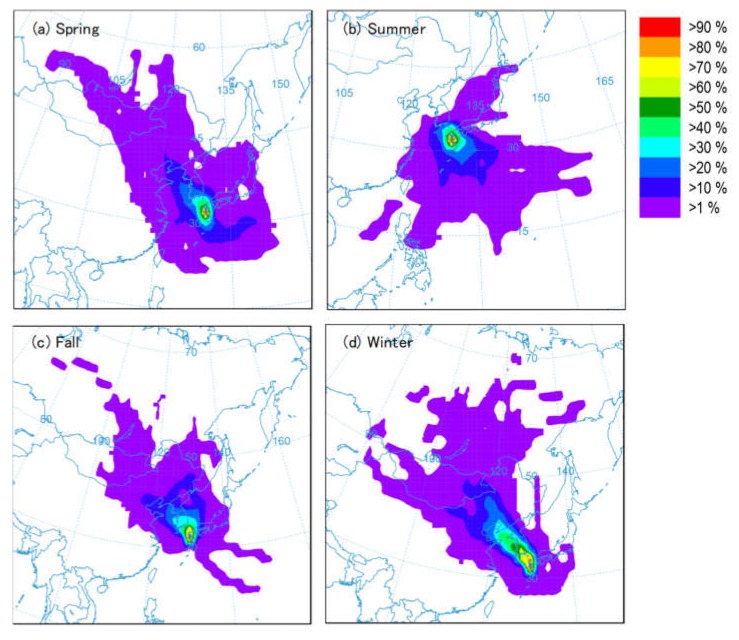
Frequency with which the back trajectories from the sampling site passed over a grid cell calculated by HYSPLIT Model of the National Oceanic and Atmospheric Administration, USA: (**a**) 29 March 2018–3 May 2018; (**b**) 29 June 2018–3 August 2018; (**c**) 29 September 2018–3 November 2018; (**d**) 30 December 2017–3 February 2018.

Figure 2 shows back trajectory analysis results for the forest site in 2018. These results were used to investigate seasonal trends relating to air mass advection. In spring, advection from the northwest was dominant, while some advection from the south side was also observed. In summer, many trajectory lines extended to the southeast side of Japan. In fall, there were many advections from the north and/or northwest sides. In winter, advection from the northwest was clearly dominant. In other words, air masses from the spring, fall, and winter seasons were transported from continental regions, such as northern China and the Korean peninsula, to the forest sampling site. Winter appeared to be strongly influenced by air masses that traveled through the continental region. Summer, on the other hand, was strongly influenced by air masses moving from the Pacific Ocean side. There appeared to be a weak influence from air masses from the continental region. Although not shown in Figure 2, similar trends were seen in other years.

### 3.2. Concentration of PAHs and Genotoxicity

Table 1 shows PAH concentration and genotoxicity measurements at both sampling sites over 6 years, from 2012–2018. The total concentration of the 15 PAHs at the forest and suburban sites were 1.495 ± 1.570 ng/m^3^ and 1.996 ± 2.209 ng/m^3^, respectively. Although the measurement target was slightly different, it tended to be about two orders of magnitude lower than the previous reports on highly-polluted areas [73,74]. The increased amount of traffic at the suburban site is thought to give it a higher PAH concentration, compared to the forest site. Comparing each season, the values in spring, fall and winter were 2.4, 2.5 and 7.9 times higher than in summer at the forest site. At the suburban site, the values in spring, fall and winter were 3.8, 3.7 and 12.3 times higher than in summer. Both sites had the following relationship: winter > fall ≒ spring > summer. The tendency to be higher in winter and lower in summer was consistent with the previous report [56,57,58]. Higher concentrations in winter might be explained by (1) increased burning of coal and biomass for thermal heating, especially in the cold northern parts of China; (2) the prevailing winter wind blowing from northwest by the stable atmospheric pressure chart pattern; (3) lower altitude for mixing of atmospheric zones in winter; (4) lower decomposition of chemical substances due to low temperatures and weak sunshine in winter; and (5) breakthrough of the PAHs with high vapor pressure from particulate phase to the gas phase due to high temperatures in the 1-week summer sampling period.

The genotoxicity of samples from the forest site (total average) was 0.16 ± 0.16 ng -4NQO/m^3^ for −S9 and 0.65 ± 0.70 ng-2AA/m^3^ for +S9. The genotoxicity of samples from the suburban site was 0.16 ± 0.12 ng-4NQO/m^3^ for −S9 and 0.84 ± 0.74 ng-2AA/m^3^ for +S9. Comparing the two sites, the suburban site was higher in the +S9 condition. The main reason for this would be the higher levels of traffic around the suburban site, as in the case of PAHs. However, in the −S9 condition, the genotoxicity of samples from both sites were almost the same. The reason for this cannot be clarified at this time. We hope to investigate this further in the future. Since ±S9 values were converted to different positive control substances, the values cannot be compared directly. Comparing each season, the values in spring, fall and winter were 1.9, 3.5 and 9.8 times higher than in summer at the forest site in the −S9 condition, and 1.0, 2.5, and 7.6 times higher in the +S9 condition. At the suburban site, the values in spring, fall and winter were 2.6, 3.5 and 6.5 times higher than in summer in the −S9 condition, and 2.8, 5.8, and 12.9 times higher in the +S9 condition. Interestingly, these seasonal ratios were similar for both genotoxicity and PAH concentration. In all conditions, it was generally winter > fall > spring > summer. The tendency to be higher in winter and lower in summer was consistent with previous Ames test results on atmospheric genotoxicity [75,76,77]. Higher genotoxicity in winter might be explained in the same way as the PAH observations. This study would be the first longitudinal report on the atmospheric genotoxicity umu test results.

### 3.3. Seasonal Fluctuation

**Figure 3:**
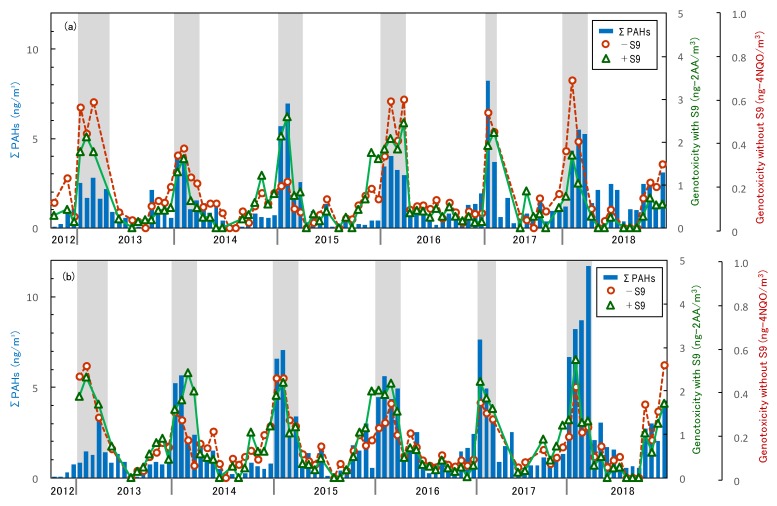
Seasonal fluctuation of PAH concentrations and genotoxicity. The bar graph represents the total PAH concentrations. The circle and triangle plots in the line graph show genotoxicity under −S9 and +S9 conditions, respectively. The gray areas indicate winter data: (**a**) values of the forest site; (**b**) values of the suburban site.

Figure 3 shows the results of continuous PAH concentration measurements and genotoxicity. Although the suburban site seemed to have higher PAH levels in the winter of 2018, there was no notable increase or decrease over the 6-year study duration. Both sites show higher PAH concentration and genotoxicity values in the winter, as indicated by the gray part of the figure. PAH concentration and genotoxicity showed the same seasonal patterns of increased levels in winter and lower levels in summer throughout the 6-year study period. This means that the PAH concentration and genotoxicity were correlated. Although they included the several effects that elevated winter concentrations and lowered summer concentrations, such as phase transition of some light PAHs, this correlation suggests that PAHs and genotoxic substances share common sources. Specifically, since there were several plots with different fluctuation trends in values even for the same sample, it was considered that there could be additional sources of genotoxic substances, other than PAHs. Despite seasonal changes in wind direction, the suburban site had almost the same seasonal fluctuation pattern and similar quantitative values as the forest site. This suggested that these areas might be affected by long-range transported pollutants, especially in winter.

### 3.4. PAH Source Identification

**Figure 4:**
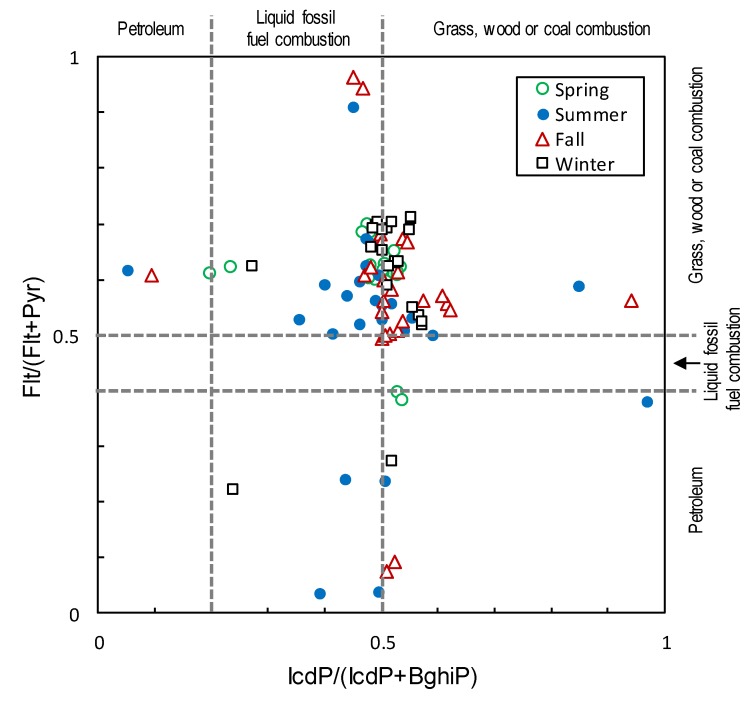
Cross plot of the diagnostic ratios of the forest site for PAH sources in each season.

**Figure 5:**
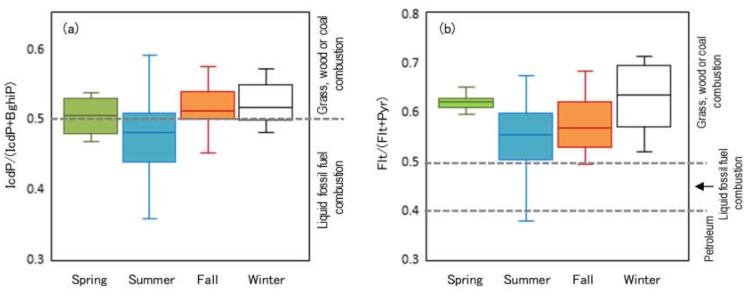
Ranges of the diagnostic ratios of the forest site for each season, excluding outliers. The upper and lower ends of the bars represent the upper and lower limits of the observed values. The upper and lower ends of the boxes represent the third and first quartiles. The lines intersecting the boxes represents the median values: (**a**) ranges of IcdP/(IcdP+BghiP); (**b**) ranges of Flt/(Flt+Pyr).

To analyze the source of the collected PAHs, Flt/(Flt+Pyr) and IcdP/(IcdP+BghiP) values were calculated using the measured values from the forest site, as shown in Figure 4. The summer plots varied relatively widely, suggesting that they were influenced by various sources for example, biomass or coal combustion, petroleum combustion, or petroleum itself. Figure 5 shows the distribution of values for each season. This was produced by statistically-processing the values on both axes in Figure 4 after removing outliers. Values exceeding 1.5 times the interquartile range were defined as outliers. In either index, the summer values tended to be lower than the other seasons. In particular, for the ratio IcdP/(IcdP+BghiP), the median value was in the area of liquid fossil fuel combustion only in summer. Considering the back trajectory analysis results, the influence of air masses from the Pacific Ocean side would be significant in summer. Ships might be a considerable source of summer PAHs. Compared to the IcdP/(IcdP+BghiP) ratio in summer, it was shown that the plots in spring, fall and winter shifted to the biomass or coal combustion side, with some variation. This may be due to the fact that PAHs would be generated from coal heating in the cold continental regions of northern China and the Korean peninsula. This is consistent with the back trajectory analysis results. According to a previous report, central heating systems are widely used in cities in north of China from October to April [78], including the spring and fall period of this study. Therefore, even in the spring and fall, the plots were considered to have shifted to the biomass or coal combustion side. The IcdP/(IcdP+BghiP) ratio in spring and fall were not much different from winter. This suggests that IcdP and BghiP were predominantly derived from biomass or coal combustion, rather than from petroleum combustion. Since the fluctuation patterns are correlated, as shown in Figure 3, it is suggested that PAHs and genotoxic substances share common sources. Therefore, biomass or coal combustion in the continental region during cold weather (especially in winter) would also be the main origin of genotoxic substances.

### 3.5. Contribution of Transboundary Pollution

At present, the impact of transboundary pollution cannot accurately be quantitatively evaluated. However, it is important to have an image of the magnitude of transboundary pollution effects. In order to estimate the extent of transboundary pollution, the forest and suburban sites were compared using the concentration of PAHs in winter (6-year average), when the impact of transboundary pollution was expected to increase. From Table 1, the concentration of PAHs at the forest and suburban sites were 3.485 ng/m^3^ and 4.761 ng/m^3^, respectively. Assuming that all the PAHs at the forest site were derived from transboundary pollution flying directly into the suburban site, the contribution of transboundary PAH pollution could reach up to 70% at the suburban site. Based on a three-dimensional chemical transport model, Ikeda et al. estimated relative contribution ratios of Northeast China, Central North China, Central South China, Korean Peninsula, and Japan to PM2.5 in the winter on Fukue Island, which is a remote island about 100 km west from the forest site. The relative contribution ratios were reported as 7.3%, 60.6%, 29.2%, 11.8%, and 1.6%, respectively [79]. Therefore, the contribution rate of 70% obtained in this study would not be unreasonable. Since the impact of transboundary pollution is considered to be at a level that cannot be ignored, it is necessary to continue related research and make international efforts to solve the problem as soon as possible.

## 4. Conclusions

- Throughout the 6 years studied, there was no notable increase or decrease in PAH concentration or genotoxicity at either the forest or suburban sites. This indicated that the air pollution around these sites caused by PAHs or genotoxic substances did not improve or worsen during this period.
- In terms of PAH concentration and genotoxicity, the suburban and forest sites shared similar seasonal fluctuation patterns and quantitative values, suggesting that the western end of Japan might be affected by long-range transported pollutants, especially in winter.
- Back trajectory analysis results strongly suggested the influence of transboundary pollution from continental regions, such as northern China and the Korean peninsula, especially in winter. Combined with analysis of the diagnostic ratios of PAHs, coal or biomass combustion in the continental region was considered one of the main sources of atmospheric PAHs at the sampling site.
- PAH concentration and genotoxicity were correlated, showing the same seasonal patterns of increased levels in winter and lower levels in summer throughout the 6-year study period. It was considered that PAHs and genotoxic substances share common sources. This suggested that transboundary pollutants caused by coal and biomass combustion from the continental region were major sources of genotoxicity.
- It was estimated that the contribution of the transboundary PAH pollution at the suburban site could reach up to 70%.

## Figures and Tables

**Table 1:** PAH concentrations and genotoxicities of aerosols collected in Nagasaki from 2012 to 2018.

Parameters	Forest Site					Suburban Site				
	Total Average	Spring	Summer	Fall	Winter	Total Average	Spring	Summer	Fall	Winter
Concentration of										
PAHs (ng/m³)	(n = 95)	(n = 21)	(n = 25)	(n = 26)	(n = 23)	(n = 97)	(n = 22)	(n = 25)	(n = 26)	(n = 24)
Acy	0.012 ± 0.021	0.004 ± 0.002	0.005 ± 0.008	0.007 ± 0.007	0.034 ± 0.034	0.024 ± 0.105	0.005 ± 0.003	0.003 ± 0.003	0.010 ± 0.012	0.079 ± 0.203
Ace	0.003 ± 0.005	0.001 ± 0.001	0.004 ± 0.007	0.004 ± 0.006	0.002 ± 0.002	0.004 ± 0.006	0.001 ± 0.001	0.005 ± 0.008	0.004 ± 0.008	0.003 ± 0.003
Flu	0.032 ± 0.086	0.006 ± 0.005	0.024 ± 0.082	0.009 ± 0.008	0.090 ± 0.140	0.046 ± 0.119	0.042 ± 0.151	0.005 ± 0.007	0.017 ± 0.036	0.125 ± 0.165
Phe	0.182 ± 0.257	0.101 ± 0.063	0.027 ± 0.037	0.081 ± 0.077	0.538 ± 0.302	0.198 ± 0.301	0.135 ± 0.082	0.046 ± 0.045	0.095 ± 0.070	0.526 ± 0.459
Ant	0.035 ± 0.162	0.015 ± 0.039	0.005 ± 0.008	0.018 ± 0.023	0.104 ± 0.321	0.047 ± 0.170	0.032 ± 0.074	0.013 ± 0.023	0.018 ± 0.025	0.129 ± 0.324
Flt	0.273 ± 0.322	0.204 ± 0.127	0.053 ± 0.053	0.198 ± 0.194	0.662 ± 0.399	0.342 ± 0.413	0.275 ± 0.155	0.047 ± 0.041	0.203 ± 0.157	0.861 ± 0.508
Pyr	0.177 ± 0.194	0.125 ± 0.063	0.054 ± 0.095	0.133 ± 0.127	0.409 ± 0.225	0.211 ± 0.229	0.173 ± 0.079	0.049 ± 0.047	0.141 ± 0.123	0.489 ± 0.281
BaA	0.065 ± 0.076	0.045 ± 0.025	0.021 ± 0.031	0.061 ± 0.061	0.136 ± 0.103	0.124 ± 0.368	0.093 ± 0.087	0.017 ± 0.014	0.061 ± 0.061	0.333 ± 0.700
Chr	0.168 ± 0.194	0.122 ± 0.084	0.034 ± 0.038	0.148 ± 0.134	0.378 ± 0.252	0.245 ± 0.296	0.178 ± 0.135	0.036 ± 0.031	0.248 ± 0.209	0.521 ± 0.412
BbF *	0.268 ± 0.390	0.161 ± 0.125	0.081 ± 0.063	0.167 ± 0.182	0.705 ± 0.600	0.467 ± 0.647	0.260 ± 0.248	0.081 ± 0.073	0.387 ± 0.429	1.173 ± 0.895
BkF *	0.051 ± 0.069	0.047 ± 0.063	0.026 ± 0.037	0.055 ± 0.037	0.079 ± 0.113	0.068 ± 0.081	0.054 ± 0.046	0.018 ± 0.016	0.065 ± 0.057	0.140 ± 0.119
BaP	0.097 ± 0.097	0.088 ± 0.086	0.035 ± 0.043	0.098 ± 0.090	0.173 ± 0.108	0.094 ± 0.098	0.087 ± 0.052	0.035 ± 0.053	0.095 ± 0.074	0.159 ± 0.144
DahA	0.023 ± 0.054	0.023 ± 0.058	0.017 ± 0.037	0.014 ± 0.011	0.038 ± 0.085	0.019 ± 0.025	0.015 ± 0.014	0.005 ± 0.007	0.022 ± 0.019	0.035 ± 0.038
IcdP	0.092 ± 0.109	0.069 ± 0.058	0.044 ± 0.057	0.095 ± 0.154	0.163 ± 0.094	0.117 ± 0.128	0.084 ± 0.069	0.025 ± 0.034	0.108 ± 0.085	0.251 ± 0.162
BghiP	0.094 ± 0.135	0.083 ± 0.087	0.040 ± 0.052	0.065 ± 0.049	0.194 ± 0.224	0.140 ± 0.190	0.109 ± 0.119	0.035 ± 0.035	0.109 ± 0.089	0.313 ± 0.286
ΣPAHs	1.495 ± 1.570	1.039 ± 0.587	0.439 ± 0.471	1.117 ± 0.795	3.485 ± 1.864	1.996 ± 2.209	1.469 ± 0.662	0.388 ± 0.283	1.436 ± 0.991	4.761 ± 2.721
Genotoxicity	(n = 81)	(n = 17)	(n = 19)	(n = 25)	(n = 20)	(n = 82)	(n = 17)	(n = 19)	(n = 24)	(n = 22)
-S9 (ng-4NQO/m^3^)	0.16 ± 0.16	0.076 ± 0.040	0.040 ± 0.043	0.14 ± 0.062	0.39 ± 0.18	0.16 ± 0.12	0.12 ± 0.048	0.046 ± 0.037	0.16 ± 0.11	0.30 ± 0.11
+S9 (ng-2AA/m^3^)	0.65 ± 0.70	0.21 ± 0.15	0.21 ± 0.22	0.53 ± 0.44	1.6 ± 0.64	0.84 ± 0.74	0.39 ± 0.21	0.14 ± 0.13	0.81 ± 0.48	1.8 ± 0.44

All data represent mean ± SD. * Since BbF and BkF had been measured as a total value at the beginning of this study, the amount of data for BbF and BkF was about 60% of other PAHs.
